# Rabies elimination in Southeast Asia: uneven progress, time to step up

**DOI:** 10.1016/j.lansea.2026.100882

**Published:** 2026-10-12

**Authors:** The Lancet Regional Health – Southeast Asia

Bhutan was recently validated by WHO for having eliminated dog-transmitted human rabies as a public health problem, setting an example for other countries in southeast Asia. Although this is an impressive feat, not all countries in the region are on the same trajectory. Rabies causes an estimated 59,000 deaths annually, with the majority occurring across Asia and Africa. The burden falls disproportionately on poor, rural, and vulnerable communities, and children younger than 15 years account for 40% of rabies mortality.

WHO has envisioned a target of zero rabies deaths by 2030. The key strategies for rabies elimination follow the One Health approach including mass dog vaccination, improved access to post-exposure prophylaxis (PEP), and community mobilisation and awareness campaigns. The recommendations on pre-exposure prophylaxis (PrEP), however, remain context dependent. In this volume, Luka and colleagues report findings from a modelling study for rabies elimination in Kerala, India—a setting with high PEP coverage. Consistent with existing evidence, the findings highlight that, over a 10-year time horizon, routine childhood PrEP prevented only ten additional deaths beyond those averted by current practice and that PreP is not cost effective. The authors recommend that PrEP be considered only once the gains achievable through strengthened dog vaccination and PEP delivery have been exhausted.

While the most effective strategies for rabies control are established, regional implementation remains fragmented. All countries have national rabies programmes, and the majority provide PEP free of charge at public hospitals. However, systematic efforts and cross-cutting coordination across stakeholders and departments are lacking. Like other infectious disease, rabies surveillance suffers from challenges like weak digital and health system infrastructure, limited laboratory capacity, lack of trained professionals and community engagement, and minimal integration with veterinary health departments. This has resulted in incomplete aggregate data across several countries of the region.

Strengthening surveillance is paramount. Countries such as Sri Lanka have developed robust surveillance and notification systems, with disaggregated data available from both dogs and humans. Similar to other notifiable infectious diseases, rabies must also be subject to stringent mandatory notifications from both the private and public sectors. Robust data capture systems that generate disaggregated estimates with integration of clinical and laboratory information conforming to standards prescribed by WHO must be devised. This could be complemented by surveillance among dogs to estimate dog rabies and identify high-risk zones for programme prioritisation.

Several countries offer valuable lessons in rabies control initiatives. Bhutan has achieved gains through a One Health approach that combines dog vaccination and dog population management with strong country-level ownership and inter-departmental coordination. Community participation has been a key driving force, with volunteers mobilising ground-level efforts. Sri Lanka and Bangladesh have similarly demonstrated strong government ownership and political commitment through country-led dog vaccination initiatives. Early outcomes from these initiatives show a positive response, with a declining incidence of human rabies deaths.

However, Myanmar, Nepal, and India remain far behind in their control efforts. Despite gross under-reporting, India continues to be one of the largest contributors to the global rabies burden. In India, while the most recent community-based modelling study estimated a human rabies death of 5726 (95% UI 3967–7350), the official sources reported less than 100 deaths a year. The usage of the Integrated Health Information Platform (IHIP) in India for rabies captures only preliminary details and does not satisfy the minimum data elements required for surveillance. In addition, surveillance is focused mostly on dog bites without follow-up information, and there is lack of data on laboratory confirmation and dog rabies.

In India, rabies control initiatives like dog vaccination and dog population control remain ad-hoc and fragmented, largely dependent on non-governmental organisations rather than sustained government programmes. Beyond rabies transmission, fatalities from dog mauling also remain poorly documented, which often leads to reactive strategies like dog culling and threatening animal welfare. Despite repeated court mandates directing the government to control dog populations, no meaningful efforts are yet in sight. Successful demonstration models have been observed in smaller states like Goa and Sikkim, largely supported through external assistance. The key barrier to progress is a lack of political commitment, government ownership, and financial support, with under-reported estimates suppressing the case for action.

This year's World Rabies Day theme of “stronger together” rightly captures the inter-sectoral collaboration missing from rabies control efforts in the region. Countries which have taken meaningful steps should sustain gains by strengthening surveillance and a multisectoral approach. For a high-burden country like India, a robust surveillance system, with upscaled centralised fiscal and infrastructural support, along with government-led dog vaccination and population management implemented at the state level, and, critically, anchored in strong community participation, is the most feasible and sustainable way forward.

